# “Don't need [therapy]! Not necessary, that's what we're for!”: Does content from fibromyalgia Facebook peer support groups emulate psychological flexibility principles?

**DOI:** 10.1016/j.pecinn.2023.100144

**Published:** 2023-03-02

**Authors:** Lyndsay Crump, Diane LaChapelle

**Affiliations:** Department of Psychology, University of New Brunswick, PO Box 4400, Fredericton, NB E3B 5ª3, Canada

**Keywords:** Fibromyalgia, Peer support, Acceptance and commitment therapy (ACT), Psychological flexibility, Facebook, Online support group

## Abstract

**Objective:**

Many persons with fibromyalgia (FM) use online peer support groups (OPSGs) to address unmet emotional or psychological needs. Some OPSG members have suggested that participation in an OPSG is a viable substitute for professional psychological services, however, no published research exploring this claim was identified.

**Methods:**

Discussion content collected from three Facebook FM OPSGs was thematically analyzed to explore whether the content posted in FM OPSGs emulated content consistent with the psychological flexibility model underlying Acceptance and Commitment Therapy (ACT) – an evidence-based psychotherapy for chronic pain conditions.

**Results:**

The content posted in OPSGs did not emulate and often contradicted the core psychological flexibility processes or skills emphasized in ACT programs.

**Conclusion:**

Participation in an FM OPSG should be approached cautiously. Content from the FM OPSGs should not be considered a substitute for professionally delivered ACT, although participation may provide emotional support to help individuals move towards readiness for active psychotherapy.

**Innovation:**

This research represents a novel application of the psychological flexibility model underlying ACT to assess the potential therapeutic value of a peer support community. Additionally, it is the first to clarify that content in FM OPSGs is not aligned with psychological flexibility processes.

## Highlights

• Posts from 3 fibromyalgia online support groups were thematically analyzed.
• The psychological flexibility model was used as an analytical framework.
• Content from the groups did not emulate psychological flexibility.
• These groups may help persons not yet ready for active psychotherapy.
• Participation in an FM OPSG should be approached cautiously.

## 1 Introduction

Fibromyalgia (FM) is a chronic, debilitating disorder affecting 1–5% of adults worldwide, the majority of whom are women [1,2]. The hallmark symptoms of FM are generalized pain, fatigue, sleep problems, mood difficulties and cognitive dysfunction that collectively hinder functioning and quality of life (QoL) [[3], [4], [5]]. Given there is no cure, FM treatment guidelines recommend incorporating education and pharmacological, exercise, and psychological therapies to improve patients' functioning and QoL [[6], [7]].

Acceptance and Commitment Therapy (ACT) is one of several evidence-based psychological treatments for pain conditions like FM that can help individuals live a full, meaningful life despite emotional distress and physical pain [[8], [9], [10]]. To do this, ACT seeks to help individuals improve their psychological flexibility, defined as the ability to accept moment-by-moment experiences without judgment and to behave in ways that align with personal values [9]. Psychological flexibility can be deconstructed into six core interacting processes (defined in italics in Table 1), which are associated with improved physical and mental health functioning, reduced medical service usage, and improved QoL among persons with chronic pain conditions [9,[11], [12]]. In contrast, psychological inflexibility, which has six processes that directly contrast with the psychological flexibility processes (see Table 1), is defined as an unwillingness or inability to adapt to new contexts due to an excessive influence of internal experiences (e.g., thoughts and feelings) and engagement in activities that do not align with personal values [9]. Psychological inflexibility is associated with increased suffering and psychopathology across a variety of health populations [9,13].

**Table 1:** Psychological flexibility/inflexibility operational definitions and coding rules.

	Psychological flexibility	Psychological inflexibility
Contact vs loss of contact with the present moment	Contact with the present moment content reflected *connection and engagement with present-focused experiences* or efforts to direct attention to the present and away from past or future.	Loss of contact with the present moment content reflected *connection and engagement with past- or future-oriented experiences*.
Cognitive defusion vs fusion	Cognitive defusion content was indicative of the ability *to observe one's thoughts without automatically believing them to be truthful, valid, or logical and without them limiting one's prospective behavioural repertoire*.	Cognitive fusion content was indicative of *entanglement with thoughts such that they are automatically accepted irrespective of their logic or veracity and increase behaviours that interfere with pursuit of a rich, meaningful life*.
Acceptance vs experiential avoidance	Acceptance content reflected *openness and willingness to experience unpleasant thoughts, feelings, or bodily sensations without trying to modify, avoid, control, or escape them*.	Experiential avoidance content reflected *refusal or unwillingness to experience unpleasant thoughts, feelings, or bodily sensations and effort to avoid, modify, or escape them.*
Self as context vs fusion with a conceptualized self	Self as context content reflected *connection with a stable, present-focused self that transcends experiences or self-descriptive thoughts and/or notices and observes both internal and external experiences.*	Fusion with a conceptualized self content reflected *attachment to a particular conceptualization of self that restricts how new experiences are interpreted* (sometimes referred to as fusion with negative self-concept).
Values vs lack of values	Values content illuminated *what truly matters or is fundamentally important and meaningful in life* to group members.	Lack of values clarity content reflected *living according to socially dictated rules, fused beliefs, or experiential avoidance*, *or* that was indicative of a *disconnection from values*.
Committed action vs unworkable action	Committed action content reflected *efforts to live according to one's values rather than socially dictated rules or expectations and despite the increased chance of encountering unpleasant thoughts, feelings, or sensations.*	Unworkable action content reflected *choosing behaviours incongruent or in conflict with personal values to avoid unpleasant thoughts, feelings, or sensations*.

Operational definition for each psychological flexibility and inflexibility pillar based on Hayes and colleagues' [9] and Harris' [28] definitions is presented in italics.

Persons with FM often encounter obstacles (e.g., lack of knowledgeable therapists within geographic limitations, financial issues) to accessing professional evidence-based psychotherapies that promote wellness and psychological flexibility [[14], [15]]. Research suggests persons with FM often seek out information and support online and that approximately 25% have participated in an online peer support group (OPSG) [16]. OPSGs may be popular within FM populations because they are available anytime, from any place with internet access, with no direct costs, and minimal perceived risk of stigmatization [17,18].

Previous exploration of content posted to Facebook FM OPSGs indicated (among other things) that persons with FM use OPSGs to address unmet mental health needs [19]. This research also suggested that *some* persons with FM may view support from peers as a potential substitute for professional mental health services [19]. If OPSGs offer similar content to that offered in an evidence-based therapy protocol, they could be recommended by peers and health providers as a cost-effective, accessible alternative to professional psychological services. If they do not, however, personal experiences within an OPSG may deter persons with FM from seeking professional psychological services that could improve their QoL. To the authors' knowledge, no published study has assessed whether content posted in OPSGs emulates content that would be delivered in an evidence-based intervention for physically and emotionally painful conditions like FM.

## 2 Methods

The authors conducted a complete re-analysis of previously collected data from three large FM OPSGs that was previously explored using an atheoretical, inductive thematic analysis [see 19]. In keeping with expert recommendations [20,21], this dataset was collected using nonparticipatory observational methodology which precluded interaction with participants. Detailed explanation substantiating the large OPSGs included in this research as public spaces (e.g., minimum 500 members, accessible by any Facebook user who clicks ‘join’) and that the conditions required to justify exemption from the general requirement of [informed] consent (i.e., minimal risk of harm or adverse effects on participants' welfare, noninteractive methodology that precluded provision of a service, public access to results via open access publication) were met in compliance with Canadian and international research ethics policies and standards and the authors' University Research Ethics Board [[22], [23], [24]] was included in the published inductive analysis [19].

### 2.1 Group selection

The data for this study came from three FM OPSGs that were originally selected from among 100 groups identified using the Facebook search option [see 19]. Groups that were non-English speaking or affiliated with a country outside North America, and those that identified a specific religious, coping (e.g., natural recovery), or healing/goal (e.g., weight loss) orientation were excluded. The authors joined the first 3 eligible groups from the original list of FM OPSGs and collected all content posted in each group over a 7-day period. For efficiency, the authors subsequently accessed archived posts from each group during the same 7-day period of the previous 2 months. Access to the archived content from the earliest designated month of data collection was not possible for one of the groups due to the magnitude of posts made in the group (i.e., data was collected for 14 days rather than 21 days for this group as attempts to go further back in the archive caused all internet browser applications used to access Facebook to crash). Approximately 600 posts and associated responses (> 33,000 kilobytes of data) posted by 1121 unique members was collected.

Due to the noninteractive nature of this study, demographic information about the group members (approximately 15,000 collectively) could only be surmised from information posted in the discussions (e.g., pronouns, discussion of menopausal or gynecological difficulties, pregnancy, mothering). Posted content suggested most participants were North American women and/or females whose illness duration (e.g., months to decades), relationship status, age (20s–70s), spirituality, and financial status varied considerably. Fewer than 10 OPSG members self-identified as male/men or as non-North America.

### 2.2 Data analysis

In accordance with the six phases of deductive thematic analysis and guidelines to ensure trustworthiness in qualitative research [[25], [26], [27]], the first author immersed herself in the dataset, which was comprised of extracted posts from the three FM OPSGs to achieve familiarity. Next, ACT was selected from the pool of viable evidence-based psychological treatment approaches for pain (e.g., CBT, mindfulness-based stress reduction) because of the authors' expertise with this approach and because ACT, which includes elements of mindfulness and CBT (e.g., emotional literacy), can significantly and positively impact the mental and physical health of persons with pain [[10], [11], [12]]. The authors utilized the published works of psychological flexibility creators Hayes and colleagues [9] and expert Harris [28] to develop a coding template that defined each process of the psychological flexibility/inflexibility model (upon which ACT is based; see Table 1). This framework was reviewed by two external clinical psychologists to ensure transparency and fidelity to the psychological flexibility/inflexibility model [26,27]. Using this coding template and NVIVO-12 software, the first author reviewed each post and response to identify text reflecting the presence of one or more psychological flexibility or inflexibility components. Initial codes were then organized to determine whether a thematic map representative of all or some components of the psychological flexibility/inflexibility model was present. Throughout this process, the first author kept an audit trail of coding decisions, reflexive thoughts, and other field notations. Discussion with the second author and research laboratory colleagues occurred throughout the research process to uphold trustworthiness criteria, to enrich the quality of the first author's interpretations, and to explore alternative interpretations of the data [[25], [26], [27]].

### 2.3 Researcher frame of reference

Both authors have completed doctoral training in clinical psychology and provide psychological services to Canadians with an array of pain conditions. Both authors also experience chronic pain.

## 3 Results

Content aligning with the six psychological flexibility processes occurred sporadically and generally elicited brief, vaguely supportive responses (e.g., posting a smiley emoji, “thank you for sharing”). In contrast, content that aligned with the six psychological inflexibility processes was posted consistently and was highly salient in all three OPSGs. Individual posts often reflected more than one inflexibility process (e.g., cognitive fusion and experiential avoidance). Inflexible content often provoked multiple responses from diverse group members that conveyed solidarity with the poster or validated aspects of psychological inflexibility. A more nuanced description organized by each flexibility and associated inflexibility process is provided below (see Table 1 for coding definitions for all processes).

### 3.1 Contact vs loss of contact with the present moment

Posts reflecting the psychologically flexible process of connecting to the present moment occurred sporadically but were positively received by others. These posts did not usually reference being present focused explicitly (e.g., “I can't just keep chasing the future all the time (a cure) without at least focusing some time and energy coming to grips with my present health circumstances …”). Responses to these posts typically included a ‘like’ or affirming emoji (e.g., smiley face).

In contrast, content indicative of loss of contact with present moment experiences was consistently observed in all groups. Group members described worrying about future-oriented events and anticipated symptoms (e.g., “I am afraid if I do anything I will be screwed tomorrow”), and longing for a less symptomatic future (e.g., “…believe there will be an end to all the suffering one day”) to the extent that some members reported it negatively impacted sleep and work functioning. They similarly described a sense of longing and chronic mental revisiting of life before FM (e.g., “I want [my life] back so I can do the things I used to do…”). Responses to posts reflecting loss of contact with present moment experiences often commiserated with the poster and reflected the responders' past or future-oriented thinking (e.g., “Depressing isn't it! I walk like an old lady due to leg pain and stiff arms. I'm only 49 but had this horrible thing since [onset year] it only seems to get worse”).

### 3.2 Cognitive defusion vs fusion

Cognitive defusion posts occurred rarely, usually in response to other members' fusion with problematic beliefs. For example, a poster described setting boundaries with a relative as “showing [her] ugly side”. Two responders pointed out that setting boundaries should not automatically be seen as ‘ugly’ or ‘selfish’ (i.e., “setting boundaries with others is NOT your ugly side”, “I've learned that I have to take care of myself… and that doing so DOES NOT make me selfish”). Based on their comments, these responders recognized cognitive fusion in the original post and offer a response that may help the poster disentangle from her fused belief. Content indicative of cognitive fusion was very prominent across the three FM OPSGs. Although not explicitly tied to time orientation (as is the case with the ‘contact/loss of contact with the present moment processes), members often described fusion or unquestioning belief in negative perceptions of past experiences or catastrophized expectations for the future. These beliefs were usually described in tandem with expressions of emotional and somatic distress and restricted behavioural responses:

Poster: “Does anyone find that after a bad pain spell the depression hits…”.

Responder: “Rest, nutrition, and lots of water, it's the only way to really get past anything.”

Responder: “A huge storm is due in a few hours. I'm worried we'll lose power… it'll be cold and that affects me…just so many stressful things going through my head… it's still stressing me to the point that I am crying and angry and upset and hurting and feel awful! You're not alone...I wish we all didn't hurt so much. Sorry for the vent on your post!”

Members similarly posted original or response content indicative of fusion with perceived helplessness or pain-related rules, negative judgments, and reasons physical or mental rehabilitation is not possible:

Poster: “I had a family member tell me if I worked on it I could do lunge exercises. I proceeded to tell her that when I do types of exercise it makes my fibro body angry and I go into a major flareup for the next couple of days. She then said I needed to do a little at a time then could do them, ya right”.

Responder: “I have the same problem. The worst person I dealt with was the lady from long term disability. She kept insisting fibro was manageable. I told her well mine's not managed and I cannot get off my couch for hours during the day!”

Responder: “Fibro is a cruel disease…unless u have fibro you don't understand the devastation this disease brings. It branches off to other diseases that don't seem to be controlled with meds. My rheumatologist says exercise is important. REALLY??? Maybe if I could get up from the sofa or chair I'm sitting on!! I'm a prisoner in my own home.”

The content of these posts and responses often conveyed high levels of perceived disability and referenced fused beliefs as a contributor to difficulties engaging in social or physical activities and increased reliance on medical interventions.

### 3.3 Acceptance vs experiential avoidance

Ideas representative of acceptance were rarely expressed and when they were, the term was typically not used explicitly. The following content from a posted meme, depicting a well-known quote by Les Brown, illustrates how the idea of acceptance was typically referenced: “It is easy to replay…how much you lost, how angry you are... you are wasting valuable time and energy that could be used to regain a new normal and start another version of your life.” This type of content generally received few responses.

Conversely, members- particularly responders- frequently offered advice (whether solicited or not) regarding how to experientially avoid rather than accept FM symptoms. This advice included reference to medication or supplements (e.g., melatonin, analgesics, marijuana), applied therapies or products (e.g., heating pad, massage), and validation of resting and avoidance of stressors, even when those stressors likely constitute valued activities (e.g., family events, recreation, social activities). Posters and responders frequently described reluctance to accept unpleasant FM-related experiences and a desire to escape FM and return to their previous ‘normal’ life (e.g., “Mowed the yard today and it took 2 hours longer than last year… Hate this disease!!”; “I wanna be NORMAL again!”; “pain makes me wants cry and crawl in a hole. Living like this sucks and I hate it. I want the old ME back”).

### 3.4 Self as context vs fusion with a conceptualized Self

Content reflecting self as context was very scarce and typically occurred in response to other members' descriptions of feeling alienated from their sense of self. For instance, in response to the post “I no longer am the person I once was,”, a peer wrote “we are the same as before you can't let a disease define you, only let it build your character and compassion for others”

On the other hand, posts and responses reflecting overattachment to or fusion with a conceptualized self were much more salient. Several versions of this inflexibility process were observed in members' posts or responses, most notably fusion with a negative, shameful FM self-concept (e.g., “worthless”, “useless”, “unattractive”, “lazy”, “weak”, “drug-seeker”, “hypochondriac”, “whiner”, “drama queen”) and fusion with FM as an identity (e.g., “we are fibro”). Fusion with the self that existed before FM and a desire to reject FM from identity was also observed regularly in the OPSG discussions (e.g., “piles of dirty clothes…late to work every other day...this isn't me. I always kept a clean house, was a model employee, energetic mother and good friend. This. Isn't. Me. I will get my life back. KEEP FIGHTING WARRIORS!”). Posts like this or responses often conveyed a sense of fusion with the “fighter” or “warrior” identity, citing a need to stoically fight against stressors like FM symptoms and perceived stigmatization (e.g., “when [doctors] say [FM] doesn't exist show them… DEMAND good service…Be the warrior. We know more about our condition than anyone else. We live it”). Collectively, fusion with these conceptualized selves was often described alongside affective distress, suicidal ideation, social withdrawal, and less openness to activity planning/pacing or assistive devices (e.g., cane). Some members also described significant distress resulting from perceived failure to embody their fused conceptualization of a ‘strong’ (stoic) self (e.g., “[family] have told me I'm strong… but I'm tired of trying to be strong because I'm in pain…I have actually been contemplating what is there to live for…I'm not strong!”).

### 3.5 Values vs lack of values clarity

Content reflecting values was uncommon. When discussed, members described a range of different personal values (e.g., family, health, autonomy) that were usually couched in content explaining increased or anticipated FM symptoms resultant from values-based activities (e.g., “I worked Thursday and then ran into town to watch my son's badminton finals. He was so happy I came so it was worth [extra pain]…”; “we gave up our tv will be going on 3 years now… my hubby and I reconnected… we're more active now [as much as] age and ability allow. Tonight we're playing poker…”). In one instance, a member posted “I will not be on [the OPSG] over the weekend because I will be celebrating with my husband” inferring that spending time partaking in an OPSG would conflict with efforts to be connected with her in-the-moment life offline. Responders to these rare posts generally conveyed validation and encouragement.

Group members sometimes described struggling to uphold socially dictated standards for financial independence, or contribution, body weight and shape, housekeeping, and relationship maintenance activities (e.g., physical intimacy) in their posts or responses but rarely clarified why these standards were valued or important to them. Members also described a sense of alienation from values like creativity or sexuality because of FM (e.g., “I am a crafter but I don't even do that anymore…now I do what is necessary then lay on the couch”) accompanied by a loss of purpose or meaning in life. Exemplifying some of these points, members responded to a post about an upcoming disability hearing stating:

Responder 1: “…. [I] just can't sit or stand to work. [I] just lay and suffer everyday. I am worth more dead than alive. At least if I died my husband wouldn't have to lose everything we have worked for.”

Responder 2: “... I feel worthless that I haven't brought any money into the house since 2011, the last time I could work”.

Posts depicting disconnection from values typically also described social withdrawal, affective distress - namely anger, despair and hopelessness –and difficulty articulating purpose or meaning in life.

### 3.6 Committed action vs unworkable action

Content depicting committed action occurred periodically, usually in response to others' posts. This content often provided an explanation for engaging or considering engaging in an activity. For instance, a responder describing how she motivates herself to be active in the morning wrote: “I want my kids not to remember me as a lump in the bed I make memories wherever I can”. Similarly, in response to a post debating whether to engage in activities that might exacerbate pain, a responder wrote, “think about what you are doing today and how you will feel tomorrow by doing it. If it is worth it, go for it!”. Generally, responses to this content were brief but supportive (e.g., “great story”, smiley emoji).

Content reflective of unworkable action was prominent and usually identified FM symptoms or interpersonal conflict as the impetus for avoidance and inaction. For example:

Poster: “I was in really bad pain from the 3^rd^ to the 12^th^… I've been not motivated to do anything… I haven't cooked or cleaned anything or even filled out my pain or food logs or the journal I was told to keep. I've been getting out of bed only when I have to go to work and going straight to bed when I get home. I haven't gone for my walk in 3 days.”

Responder: “I'm exactly the same. Now I have a broken wrist on top of it.”

Often, group members described prioritizing efforts to eradicate or avoid distress and, as a result, struggling to pursue activities that aligned with values and a life perceived to be meaningful:

“… do nothing but lay in bed moaning… finding a heating pad and crashing. I understand we have to compromise our lives to stay on a somewhat even keel, but … I have so many things I think about making. Or to go somewhere. But… diarrhea can strike with no notice and I could end up crying in public. I wanna be NORMAL again!”

Generally, posts and responses that described unworkable action also described passive coping (e.g., resting, avoidance of stressors), emotional distress, and social and relationship difficulties.

## 4 Discussion and conclusion

### 4.1 Discussion

Results of this deductive thematic analysis indicated content posted in the three FM OPSGs aligned more saliently with the psychological inflexibility processes than psychological flexibility processes or skills. No notable differences were identified between the thematic maps of each FM OPSG. Thus, individuals encountered roughly the same type of content in all three groups. This suggests FM OPSG members are more likely to encounter content that aligns with a psychologically inflexible approach to living with FM than a psychologically flexible one regardless of which group they join. This is troubling because psychological inflexibility has been consistently associated with negative patient outcomes for persons with chronic pain and psychological difficulties [9,[11], [12],^30^].

#### 4.1.1 Why was psychologically inflexible content prominent?

A post hoc literature review spanning the broader areas of adjustment to chronic illness, chronic pain, mental health, and group interventions was conducted to help illuminate why psychological inflexibility was so prominent in the OPSG discussions. This review suggested a combination of individual characteristics and group mechanisms likely contribute to the prominence of psychologically inflexible content in the FM OPSG discussions examined.

Use of nonparticipatory observation meant it was not possible to assess each member's levels of flexibility/inflexibility. However, Relational Frame Theory (the theory associated with ACT's psychological flexibility/inflexibility model) suggests an individual's expressive language may reflect their internalized psychological flexibility/inflexibility [9]. Thus, it is possible that the group members posting content in the OPSGs included in this research possessed a high internalized level of psychological inflexibility. This high internalized inflexibility may result from lack of opportunities to learn and practice psychologically flexible approaches to living with FM, social and cultural pressures to ‘cure’ rather than accept sickness or disability, exiting psychopathology (e.g., depression), or from the persistent strain imposed by FM [5,9]. Stigmatization undoubtedly bolsters psychological inflexibility by limiting the availability of offline supports and reinforcing experiential avoidance and fused, negative self-beliefs [11,[30], [31], [32], [33]]. Individuals (including those with FM) who lack psychological flexibility skills are inherently more vulnerable to emotional distress and psychological problems and, therefore, may be more inclined to seek emotional support and information about how to *fight* (i.e., experientially avoid) FM [9,28,30,33]. FM OPSGs are more accessible than professional health services and perhaps more palatable to persons with FM because of the validation, empathy, and symmetrical power distribution endogenous to peer-to-peer relationships [34]. Thus, psychologically inflexible persons with FM may be more inclined to use this type of resource to address their unmet needs, resulting in the prominence of psychological inflexibility in FM OPSG discussions.

In contrast, those who possessed or have learned to practice psychological flexibility (which is associated with positive patient outcomes and improved overall wellbeing) would be expected to be more resilient to FM-related stressors and have less need for peer support [11,35]. They may also be less inclined to engage in unproductive sense-making (e.g., efforts to avoid uncertainty or incoherence, overthinking) in an illness-specific OPSG [36]. This subgroup of individuals with FM may therefore be less inclined to participate in a FM OPSG resulting in the underrepresentation of psychologically flexible content on the groups' walls. Finally, members may be more inclined to seek support or post in the OPSG when distressed [9,11]. In this case, the OPSGs' wall posts may be biased towards capturing moments of psychological inflexibility in members lives.

Facets of the OPSG may also unintentionally cultivate content reflective of psychological inflexibility. All three OPSGs included in this research indicated that members must be *kind* and *supportive* to one another in their rules or ‘netiquette’. Given the highly stigmatized nature of FM, these rules were likely imposed to protect group members from being criticized or further stigmatized within a presumedly safe peer-to-peer environment [37,38]. Unintentionally, this requirement may discourage group members from posting some types of flexible content (e.g., encouragement to shift attention to the present rather than to past/future-oriented events; alternatives to unworkable action) or challenging fused beliefs or experiential avoidance because of fear it could be perceived as invalidating, oppositional, or otherwise unsupportive [[37], [38], [39]]. By offering well-intentioned validation or support for inflexible behaviours (e.g., avoidance), however, members may unintentionally encourage others' inflexible practices [39].

Concern about being perceived as unsupportive are not unfounded; previous research has indicated that persons with FM often perceive alternative coping suggestions as invalidating and stigmatizing [40,41]. Corroborating this point, we observed that group members sometimes posted contentious responses to comments challenging psychological inflexibility. Chronic stigmatization and invalidation likely contribute to this sensitivity as well as the pronounced need for emotional support (e.g., validation, empathy, acceptance) noted the OPSG discussions [40,41]. Under these circumstances, it is possible persons with FM use OPSGs to address deficits in basic emotional support that, until remedied, act as a barrier to readiness to address psychological inflexibility [19]. That is, responses and or suggestions that convey psychologically flexible practices may exceed the degree of change persons in FM OPSGs are ready for and consequently be perceived as invalidating.

The structure of OPSGs and the time group members invest to formulate their posts or responses might also unintentionally promote psychologically inflexible content. We observed that members often described, for example, fusion with negatively valanced perceptions of past or future-oriented experiences. While writing about these experiences, group members must inherently direct attention to the past or future which hinders contact with moment-to-moment offline experiences. Notification that others have responded to their post or comment (a feature on Facebook groups) may further encourage members to disengage from their offline life and engage with others' input on their past- or future-oriented experience, recapitulating loss of contact with the present moment. These posts also appear to prompt other group members to revisit and post about their own similar examples and fused cognitions. Thus, the structure and mechanisms that make FM OPSGs accessible and popular may also inadvertently cultivate posts reflective of psychologically inflexible practices.

Overall, results of this research suggest the FM OPSGs examined, and potentially other OPSGs, may not represent an ideal support for persons attempting to develop psychological flexibility in their lives and should not be considered a substitute for professionally delivered ACT. FM OPSGs may, however, serve an altogether different and valuable purpose for some individuals with FM. Because FM OPSG members are not required to participate in group discussions and are not held accountable for adopting or practicing skills [39,43], FM OPSGs may be a better fit for persons not yet ready for active psychotherapy. That is, FM OPSGs may be better poised to provide foundational emotional support to persons in the early stages of change (i.e., precontemplation, contemplation, and determination) who may not be ready to engage in the active work associated with an evidence-based psychotherapy like ACT [[42], [43], [44], [45]].

#### 4.1.2 Limitations and future directions

Due to the observational methodology, collection of demographic information or assessment of members' internalized degree of psychological flexibility/inflexibility was not possible. The linguistic theory associated with psychological flexibility (i.e., Relational Frame Theory), however, indicates the language individuals use (or post) may represent their internalized degree of psychological flexibility/inflexibility [9]. The observational methodology also precluded direct assessment of whether or to what degree FM OPSG participation impacts members' offline functioning and emotional wellbeing, or whether members' posted content aligned with the psychological flexibility processes outside of the data collection periods.

Analysis of similar datasets based on other psychological interventions with established or growing empirical support (e.g., Cognitive-Behavioural Therapy, Attachment-Based Compassion Therapy) could yield similar or disparate results of interest to pain investigators and clinicians [10]. Similarly, it is possible FM OPSGs with more abundant psychologically flexible content exist but were not included in this study. Future research exploring these limitations as well as group members' perceptions of the costs and benefits of OPSG participation would help determine whether FM OPSGs help or hinder positive adjustment to FM.

Research exploring whether persons with FM might benefit from participation in a Facebook OPSG moderated by an ACT-trained provider instead of (or in addition to) participation in a peer-moderated online group is warranted. If participants' emotional support needs are sufficiently met, it is possible they may be able to engage in a professionally moderated ACT-based Facebook FM OPSG designed to demonstrate, educate, and empathically address the mental and behavioural health symptoms often experienced by persons with FM. If successful, this type of professionally led OPSG may represent a novel, accessible, cost-effective resource for this underserviced clinical population. Given the overwhelming strain currently placed on health resources, research exploring the potential value of professionally led OPSGs utilizing accessible, user-friendly platforms like Facebook is strongly recommended.

### 4.2 Innovation

Although extant research indicates persons with FM often use OPSGs and perceive them to be helpful, no previous research (to the authors' knowledge) has explored whether the content available in a FM OPSG emulates that provided in an evidence-based psychotherapeutic intervention like ACT. This is problematic, because research suggests patients may view peer support and psychotherapy as synonymous [19]. Therefore, this research is specifically innovative because it represents the first published effort to utilize ACT's theoretical model (psychological flexibility) as a tool to assess the potential psychotherapeutic value of a FM peer support group (i.e., whether content is analogous to that provided in an ACT program).

The resources required to ensure this research was conducted ethically and to analyze a large, organically occurring, text-based dataset has likely contributed to the scarcity of nonparticpatory observational research on FM online support communities [[18], [19], [20],^47^]. Aside from this study's inductive precursor [19], no other research to the authors knowledges has examined a Facebook-based FM OPSG. The authors therefore posit that this study represents a generally innovative application of nonparticipatory observational methods to the Facebook FM OPSG environment. Use of this approach arguably improved the authenticity of the data and capitalized on already existing, patient-led rather than researcher-driven discussions. This reduced the burden placed on persons with FM to participate in a time-intensive and potentially emotionally and physically draining survey or interview.

### 4.3 Conclusion

These findings suggest that, for those who identify improved psychological flexibility or acceptance as an illness management goal, participation in an FM OPSG should be approached cautiously or perhaps discouraged. The results of this study will ideally prove useful to patients who are actively using or considering joining an FM OPSG and to health providers whose patients may be participating in an FM OPSG. These findings will also ideally help health providers better understand what patients may encounter in OPSGs and how it may conflict with the goals of an evidence-based psychotherapy program for persons with pain conditions (i.e., ACT). Given the popularity of FM OPSGs, these findings will hopefully encourage providers to initiate discussions about the pros and cons of FM OPSGs with their patients to help patients make informed choices about whether to join or participate in such groups.

## Funding

The author(s) disclosed receipt of the following financial support for the research, authorship, and/or publication of this article: This work was supported by the 10.13039/501100000024Canadian Institutes of Health Research [CGS-D grant to the first author], the New Brunswick Innovation Foundation Doctoral Scholarship [first author], and the Harrison McCain Foundation Young Scholar Award [second author].

## Declaration of Competing Interest

Dr. Crump declares that she has no conflict of interest. Dr. LaChapelle declares she has not conflict of interests.
